# Effects of methotrexate on Wnt/β‐catenin signaling and glial activation in temporomandibular joint arthritis in rats

**DOI:** 10.1111/eos.70041

**Published:** 2025-09-26

**Authors:** Ana Carolina de Figueiredo Costa, Luane Macedo de Sousa, Sofia Tavares Bessa, Anamaria Falcão Pereira, Paula Goes, Mariana Lima Vale, Delane Viana Gondim

**Affiliations:** ^1^ Postgraduate Program in Dentistry Faculty of Pharmacy Dentistry and Nursing Federal University of Ceará Fortaleza Ceará Brazil; ^2^ Postgraduate Program in Morphofunctional Sciences Faculty of Medicine Federal University of Ceará Fortaleza Ceará Brazil; ^3^ Dentistry Course Faculty of Pharmacy Dentistry and Nursing Federal University of Ceará Fortaleza Ceará Brazil; ^4^ Postgraduate Program in Pharmacology Faculty of Medicine Federal University of Ceará Fortaleza Ceará Brazil

**Keywords:** disease animal model, glial cells, inflammation, temporomandibular joint, trigeminal pathway

## Abstract

Rheumatoid arthritis is a chronic autoimmune disease that can affect the temporomandibular joint (TMJ) and cause pain. Methotrexate is widely used to control disease progression, but its effects on pain mechanisms are not fully understood. This study evaluated the antinociceptive effects of methotrexate on pain behavior, Wnt/β‐catenin pathway expression, and glial activation in the trigeminal nociceptive pathway in rats with TMJ arthritis. Eighteen male Wistar rats were assigned to one of three groups: control, arthritic, or arthritic treated with methotrexate. Arthritis was induced by intra‐articular injection of methylated bovine serum albumin. Pain behavior, inflammation, and joint damage were assessed. Immunoexpression of Wnt/β‐catenin components and c‐Fos was analyzed in the trigeminal ganglion and subnucleus caudalis, along with microglial activation. Methotrexate‐treated arthritic rats showed increased pain threshold, reduced facial pain behaviors, and decreased inflammatory infiltrates, along with lower levels of tumor necrosis factor‐α and interleukin‐17 in the joint compared to untreated arthritic rats. Methotrexate also reduced c‐Fos, Wnt‐10b, β‐catenin, and glutamine synthetase expression in the trigeminal ganglion and attenuated microglial activation in the subnucleus caudalis. These results indicate that methotrexate produces antinociceptive effects by modulating Wnt/β‐catenin signaling and reducing glial activation in the trigeminal nociceptive pathway in rats with TMJ arthritis.

## INTRODUCTION

Rheumatoid arthritis is a chronic, refractory autoimmune disease characterized by synovial inflammation and proliferation, leading to cartilage erosion and subchondral bone destruction. Its pathogenesis involves both innate and adaptive immune responses, driven by a complex network of inflammatory mediators and dysregulated signaling pathways [1].

Advances in understanding the signaling pathways involved in the pathogenesis of rheumatoid arthritis have significantly improved knowledge of its complex mechanisms and facilitated the development of targeted treatments [2]. Abnormal signaling of the canonical Wnt pathway has been implicated in its pathogenesis [3, 4]. Specifically, the Wnt/β‐catenin pathway contributes to nociception, synovial inflammation, and joint damage in the temporomandibular joint (TMJ) of arthritic rats [1, 5]. Elevated Wnt‐10b and β‐catenin expression in neurons and glial satellite cells, respectively, within the trigeminal ganglion of arthritic rats, further highlights its role in the trigeminal nociceptive pathway [6].

Methotrexate is the first‐choice drug for controlling rheumatoid arthritis progression. In addition to its antiproliferative, antimetabolic, and anti‐inflammatory effects, it also reduces joint damage by regulating signaling pathways such as Janus kinase/signal transducer and activator of transcription, nuclear factor kappa‐light‐chain‐enhancer of activated B cells (NF‐κB), p38 mitogen‐activated protein kinase (p38 MAPK), receptor activator of NF‐κB/RANK Ligand/Osteoprotegerin (RANKL/RANK/OPG), and Wnt signaling pathway mediated by β‐catenin (Wnt/β‐catenin) [7, 8]. Methotrexate has been shown to promote bone protection in the knee of rats with collagen‐induced rheumatoid arthritis by regulating genes associated with the Wnt/β‐catenin pathway [7]. However, to the best of the author's knowledge, the use of methotrexate for pain control in arthritis has not yet been investigated in experimental models involving the TMJ. Therefore, this study aimed to evaluate nociceptive behavior, joint damage, and the immunoexpression of the Wnt/β‐catenin pathway and glial cell markers in the trigeminal nociceptive pathway of arthritic rats treated with methotrexate in a TMJ arthritis model induced by methylated bovine serum albumin (mBSA).

## MATERIAL AND METHODS

### Animals

This study was approved by the Ethics Committee on the Use of Animals of the Federal University of Ceará with protocol number no. 148/17. All experiments were conducted following the Guide for the Care and Use of Laboratory Animals (USA National Institute of Health Publication No. 80‐23, revised 1996) and the Animal Research: Reporting of In Vivo Experiments (ARRIVE) guidelines.

Eighteen male Wistar rats (7–8 weeks old; 180–220 g) from the Central Animal Facility of the Federal University of Ceará were randomly allocated, using a computer‐based random order generator to, one of three interventions: none (control), induction of TMJ arthritis (RA), or induction of TMJ arthritis subsequently treated with methotrexate (RA + MX). The sample size was calculated to detect a statistically significant difference of 20% in joint inflammation and damage between the treated and untreated arthritic groups, with a statistical power of 80%. Consequently, six animals were allocated to each experimental group. They were housed in microisolator cages with three rats per cage, maintained at a controlled temperature of 25 ± 2°C and a 12‐h light/dark cycle, with easy access to water and food.

### TMJ arthritis induction protocol

Initially, the animals were sensitized to the antigen through subcutaneous injections on their backs. The injections consisted of an emulsion containing 500 µg of mBSA (Sigma‐Aldrich), 100 µL of phosphate‐buffered saline (PBS), and 100 µL of Complete Freund's Adjuvant or Incomplete Freund's Adjuvant (F5881 and F5506, respectively; Sigma‐Aldrich). These injections were administered on Days 0, 7, and 14 of the experiment. Subsequently, arthritic animals received intra‐articular challenges in the left TMJ with a solution of 10 µg of mBSA dissolved in 10 µL of PBS on Days 21, 28, and 35. Control animals received injections of PBS in the left TMJ [5].

In the RA + MX group, methotrexate (Tecnomet, Zodiac) was administered at a dose of 0.75 mg/kg via oral gavage twice a week, beginning 24 h after the first intra‐articular injection at Day 21 and continuing until the final day of TMJ arthritis induction (Day 35) in the RA + MX group, thereby leading to a total of four doses. The control and RA groups received only saline solution via oral gavage [9].

Euthanasia was performed on Day 36, 24 h after the third intra‐articular challenge. Animals were first anesthetized with ketamine (80 mg/kg; Vetanarcol, König) and xylazine (8 mg/kg; Kensol, König) before undergoing transcardiac perfusion with 0.9% saline and 4% paraformaldehyde (1.00496, Sigma‐Aldrich) in 0.1 m PBS.

### Assessment of nociceptive behavior

Mechanical hyperalgesia in the left TMJ was evaluated using the electronic Von Frey test (Von Frey Digital, Insight). To minimize non‐specific aversive responses, animals that had been randomly allocated to the experimental groups were acclimated to the mechanical hyper‐nociception test for five consecutive days. During each testing session, they were placed in plastic boxes for 20 min to allow habituation before stimulation. The device was then applied to the left TMJ region until the animal displayed a reflexive response, such as head withdrawal or vocalization [6]. This procedure was performed three times at the 6th hour following each of the first, second, and third intra‐articular injections by a calibrated examiner. The force intensity was recorded in grams (g), and the average of the three measurements was calculated.

Spontaneous nociceptive behavior was assessed using the Grimace Rat Scale [10]. Weekly, the animals were placed in acrylic cubes measuring 21 × 10.5 × 9 cm^3^ and filmed for 30 min using two digital cameras positioned on either side of the cubes to ensure optimal footage quality. Behavioral parameters, including orbital tightening, nose flattening, and changes in the ears and vibrissae, were evaluated for each animal. Each parameter was scored on a scale from 0 to 2, where 0 indicated high confidence in the absence of the action unit, 1 indicated high confidence in moderate appearance or uncertainty regarding presence or absence, and 2 indicated clear appearance of the action unit with high confidence. The median as well as the minimum and maximum scores were used to summarize the data for each group.

### Assessment of inflammation and joint damage

After euthanasia, the left TMJs were dissected, fixed in 10% buffered formaldehyde for 48 h, and demineralized in 4% ethylenediamine tetraacetic acid solution. The samples were then embedded in paraffin, sectioned into 5 µm‐thick slices, mounted on glass slides, and stained with either hematoxylin–eosin (H&E) or 4% toluidine blue for evaluation under an optical microscope (Leica DM 2000). For each staining protocol, three nonconsecutive histological sections from each TMJ were analyzed.

For the histopathological analysis with H&E staining, the left TMJ of each animal was evaluated. Scores were assigned to evaluate the inflammatory infiltrate in the synovial membrane as follows: 0 = absence of inflammatory infiltrate; 1 = mild infiltrate; 2 = moderate infiltrate; and 3 = intense infiltrate. The scoring was based on cell influx into the synovial membrane and periarticular tissue, as well as synovial membrane thickness. Joint damage in the cartilage was evaluated based on fibrocartilage thickness and the extent of cartilage erosion, using the following scale: 0 = no joint damage; 1 = mild damage; 2 = moderate damage; and 3 = severe damage [5]. The median as well as the minimum and maximum scores were used to summarize the data for each group.

Proteoglycan content was assessed using toluidine blue staining, which highlights metachromatic regions of fibrocartilage. This method is commonly employed to detect cartilage degradation in arthritic rat models [11]. In healthy rats, the articular cartilage of the TMJ is organized into four distinct layers: the articular zone, a zone of undifferentiated cells, fibrocartilage, and an underlying layer of calcified cartilage. Under inflammatory conditions, a reduction in the dark blue metachromatic staining of the non‐calcified layers signifies proteoglycan loss and degradation of cartilage, typically due to synovial pannus invasion or degenerative processes associated with the synovial fluid [11].

Cartilage erosion and proteoglycan depletion were evaluated in toluidine blue‐stained sections at 200× magnification. For quantitative analysis, the full region of cartilage tissue was manually delineated. Fibrocartilage thickness (in micrometers) was then measured morphometrically by a single, trained examiner (LMS) using imagej software (NIH). Three histological sections per joint were analyzed, and the mean thickness was calculated for each specimen [11].

For immunohistochemical analysis, samples were sectioned into 3 µm slices and mounted on slides coated with poly‐l‐lysine. The streptavidin–biotin–peroxidase method was used to evaluate the immunoexpression of TNF‐α, IL‐17, and IL‐10 in the synovial membrane and articular cartilage.

Following deparaffinization, rehydration, and antigen retrieval with citrate buffer (pH 6.0) for 30 min at 85°C, slides were treated to block endogenous peroxidase activity with 3% hydrogen peroxide diluted in PBS for 30 min. After rinsing with PBS, the samples were incubated overnight with primary rabbit antibodies against TNF‐α (Abcam, ab307164), IL‐17 (Santa Cruz Biotechnology, sc7927), and IL‐10 (Abcam, ab9969) at dilutions of 1:100, 1:100, and 1:150, respectively.

After primary antibody incubation, secondary antibody incubation was performed using the Envision Plus‐HRP system (Dako, K4065) or the ImmunoCruz ABC kit (Santa Cruz Biotechnology, sc‐516216), followed by immunostaining with 3,3‐diaminobenzidine (DAB) (Dako, S1961) and counterstaining with Harris hematoxylin. The sections were then dehydrated through a graded series of alcohols, cleared in xylene, and mounted with Entellan (Merck, 107960) under a coverslip.

The quantification of immunoreactive cells was performed by a trained examiner (A.C.F.C.) using an optical microscope (Leica DM 2000) at 400× magnification. Five representative fields (hot spots) were selected per sample from both the articular cartilage and the synovial membrane. These hotspots were defined as the areas exhibiting the highest density of positively stained cells, identified under low magnification and at 200× magnification. Immunopositive cells, identified by the characteristic brown staining in the cytoplasm and/or nucleus, were manually counted using imagej software (NIH). The results were expressed for each sample as the number of positive cells per microscopic field at 400× magnification.

### Immunoexpression in the trigeminal nociceptive pathway

After euthanasia, the trigeminal ganglion and the caudal part of the spinal trigeminal nucleus (Sp5C region) were dissected. These regions correspond to the location of the cell bodies of peripheral neurons and the site of the first synapse in the trigeminal nociceptive pathway, respectively. The Sp5C region was identified, and sections were cut from the area between bregma −14 mm and −15 mm. The dissected structures were placed in 4% paraformaldehyde for 2 h and cryoprotected in 30% sucrose solution for 72 h. After this period, the tissues were embedded in Tissue‐Tek (Tissue‐Tek OCT Compound, Interprise) and stored in a freezer at −80°C.

For immunofluorescence assays, sections (10 µm) were fixed in methanol (Vetec Química Fina) for 2 min and antigen retrieval was performed in 0.1 M citrate buffer (pH 6.0) for 15 min at 95°C. Next, non‐specific bonds were blocked with 0.1% Triton X‐100 (Sigma‐Aldrich) for 10 min, and the nuclear membrane was permeabilized with 0.3 M glycine (Sigma‐Aldrich) in 5% bovine serum albumin (Sigma‐Aldrich) for 30 min.

The samples were incubated overnight, at a temperature of 2–8°C, with the primary antibodies. The samples of trigeminal ganglion were incubated with anti‐cellular proto‐oncogene (c‐Fos) at a dilution of 1:200 (anti‐rabbit, 31254, Cell Signaling Technology), anti‐glutamine synthetase at a dilution of 1:200 (anti‐mouse, Merck Millipore, MAB302), anti‐Wnt‐10b at a dilution of 1:200 (anti‐rabbit, Abcam, ab70816), and anti‐β‐catenin at a dilution of 1:250 (anti‐rabbit, Abcam, ab6302).

Meanwhile, the samples of Sp5C region were incubated with anti‐c‐Fos at a dilution of 1:200 (anti‐rabbit, Cell Signaling Technology, 2250), anti‐ionized calcium‐binding adapter molecule 1 (Iba‐1) at a dilution of 1:200 (anti‐rabbit, Cell Signaling Technology, 17198), anti‐Wnt‐10b at a dilution of 1:200 (anti‐rabbit, Abcam, ab70816), and anti‐β‐catenin at a dilution of 1:250 (anti‐rabbit, Abcam, ab6302).

Subsequently, tissue sections were incubated with secondary anti‐IgG antibody Alexa Fluor 546 (goat anti‐rabbit, Thermo Fisher Scientific, A‐11010) or Alexa Fluor 633 (goat anti‐mouse, Thermo Fisher Scientific, A‐21052), both at a dilution of 1:400, for 1.5 h. This was followed by incubation with anti‐NeuN antibody conjugated with Alexa Fluor 488 (Merck Millipore, ABN78A4), at a dilution of 1:150, for 1.5 h to label neuronal bodies. Nuclear staining was performed using 4′,6‐diamidino‐2‐phenylindole (DAPI, Thermo Fisher Scientific, 3143066) (4 µL in 200 mL PBS) for 30 min. Slides were mounted using Prolong Gold Antifade Mountant (Thermo Fisher Scientific, P36930). Photomicrographs were acquired using a confocal laser scanning microscope (Zeiss LSM 710, Carl Zeiss) under 200× magnification (20×/0.8 objective), with standardized “master gain” and “digital offset” parameters.

The quantification of fluorescent areas was performed using image analysis software (fiji imagej, NIH) by an experienced examiner (AFP). Fluorescent regions in the photomicrographs were identified based on pixel color saturation, with red or green hues indicating fluorescence. Pixel selection was determined using predefined color threshold settings, established by setting upper and lower saturation limits. For c‐Fos, Wnt‐10b, and β‐catenin, quantification results were expressed as percentages, representing the proportion of anti‐c‐Fos and anti‐Wnt‐10b fluorescence co‐localized with anti‐NeuN, and β‐catenin fluorescence relative to anti‐glutamine synthetase labeling [6]. In contrast, for glutamine synthetase and Iba‐1, results were expressed as absolute cell counts, determined by counting cells showing co‐localized fluorescence for anti‐glutamine synthetase or anti‐Iba‐1 with anti‐DAPI [12].

### Analysis of microglial/macrophage morphology

To evaluate microglial and infiltrating macrophage morphology, confocal microscopy images of tissues labeled with anti‐Iba‐1 were acquired using the maximum pinhole aperture. The images were subsequently converted to 8‐bit grayscale format. These grayscale images were then binarized using appropriate image processing software (fiji imagej, NIH). After binarization, the images were processed with the Skeletonize plugin to analyze the microglial/macrophage architecture. The average number of branch end points and the total branch length per cell were calculated [13].

### Statistical analysis

Statistical analysis and graph construction were performed using graphpad prism 7 software (graphpad). The normality of the variables was assessed using the Shapiro–Wilk test, and homogeneity was evaluated with the Levene test. Parametric and homogeneous data are presented as mean ± standard deviation and were compared between groups using one‐way analysis of variance, followed by Tukey's post‐test. Non‐parametric data are presented using the median value as well as the minimum and maximum and were compared using the Kruskal–Wallis test, followed by Dunn's post‐test. A 95% significance level was adopted, and differences were considered statistically significant at *p* < 0.05.

## RESULTS

### Analysis of inflammation and joint damage

**FIGURE 1:**
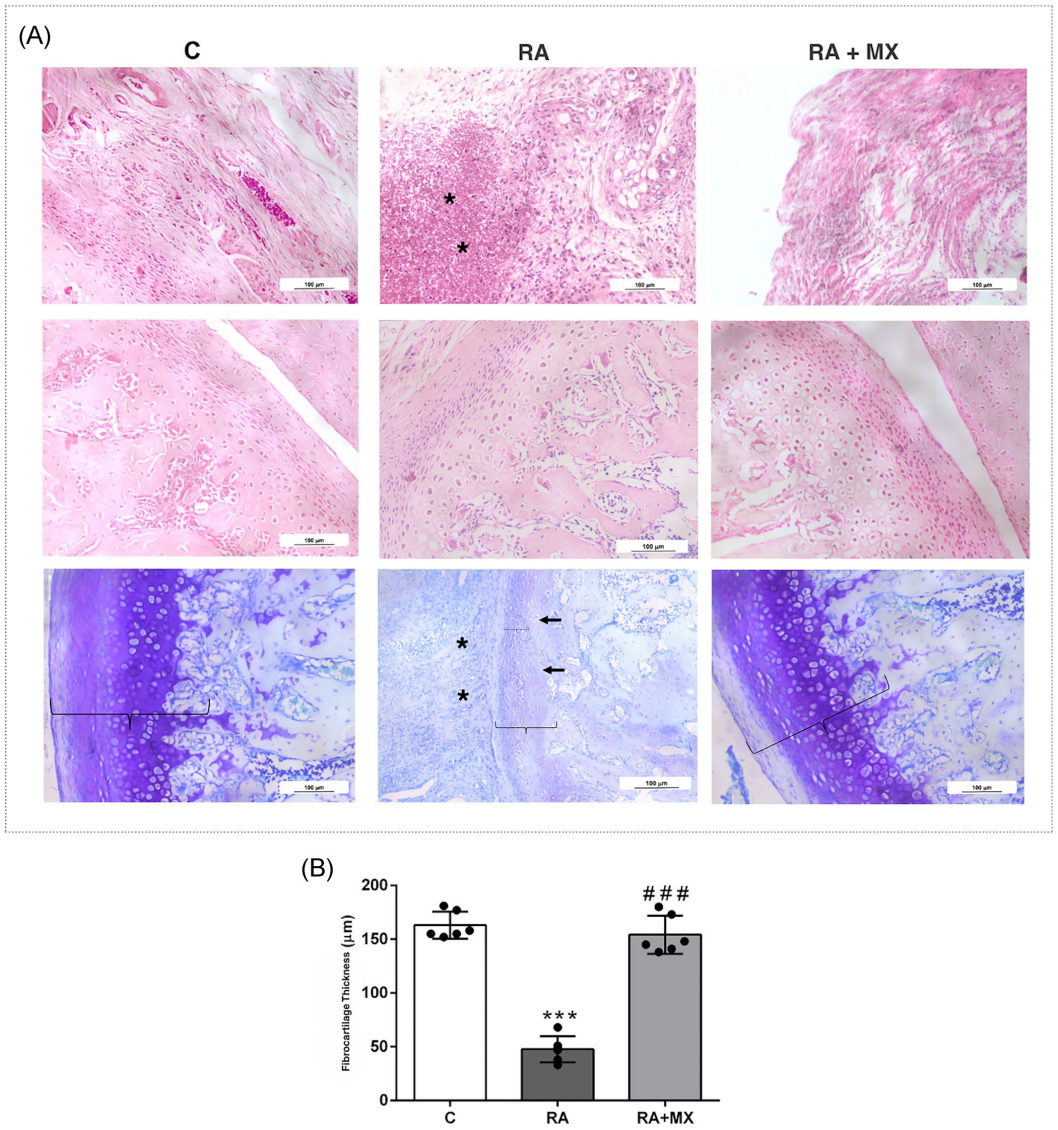
(A) Effect of methotrexate (0.75 mg/kg; twice weekly, orally) on inflammatory cell infiltration in the synovial membrane and articular cartilage thickness in animals with temporomandibular joint (TMJ) arthritis (*n* = 6). Photomicrographs of the synovial membrane (hematoxylin–eosin [H&E] staining) and articular cartilage (H&E and Toluidine Blue staining) at 200× magnification. (B) Quantitative analysis of cartilage thickness (µm) in animals with TMJ arthritis treated with methotrexate (*n* = 6). Data are presented as mean ± standard deviation (SD) (error bars represent SD). ***p < 0.0001 versus control (C); ###*p* < 0.0001 versus RA group (one‐way analysis of variance [anova] followed by Tukey's post hoc test). C = control group; RA = untreated animals with TMJ arthritis; RA + MX = animals with TMJ arthritis treated with methotrexate. Asterisk indicates inflammatory infiltrate; arrow indicates articular damage; brace indicates cartilage thickness.

Unlike the control animals, arthritic animals (RA) exhibited an inflammatory infiltrate in the synovial membrane (*p* = 0.0002) and joint damage (*p* = 0.023), accompanied by significant depletion of proteoglycans in the articular cartilage (Figure 1A,B; Table S1). In contrast, arthritic animals treated with methotrexate showed less joint damage (*p* = 0.02), with no statistically significant differences in the synovial membrane from that seen with either the control or RA animals (Table 1). Moreover, untreated arthritic animals displayed a marked reduction in joint thickness relative to the control animals (*p* < 0.0001). Methotrexate treatment maintained joint thickness at the same level as seen for controls (Figure 1B).

**FIGURE 2:**
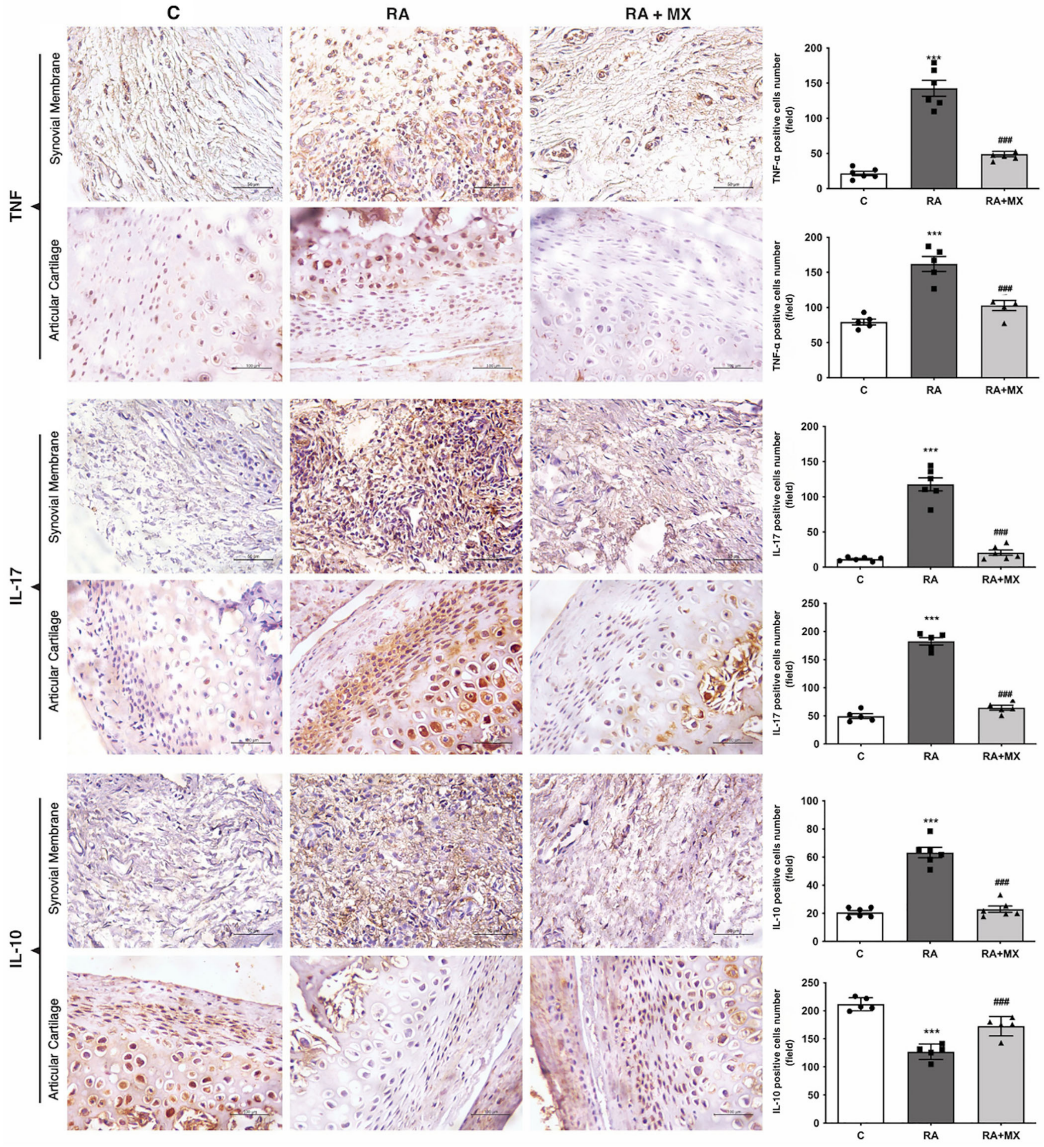
Effect of methotrexate (0.75 mg/kg; twice weekly, orally) on the expression of TNF‐α, IL‐17, and IL‐10 in the synovial membrane and articular cartilage in animals with temporomandibular joint (TMJ) arthritis (*n* = 6; 200× magnification). Graphs are presented as mean ± standard deviation (SD) (error bars represent SD). ***p < 0.001 versus C. ###*p* < 0.001 versus RA (one‐way analysis of variance [anova]; Tukey). C = control; RA = animals with TMJ arthritis; RA + MX = animals with TMJ arthritis treated with methotrexate.

Inflammation and joint damage were further evaluated by analyzing the immunoexpression of TNF‐α, IL‐17, and IL‐10 in the TMJ of arthritic animals treated with methotrexate. Untreated arthritic animals showed significantly higher levels of the pro‐inflammatory cytokines TNF‐α and IL‐17 in both the synovial membrane (mean value of positive cell count = 142 and 118, respectively) and the articular cartilage (positive cell count means = 162 and 182, respectively) than did the non‐arthritic control animals and the methotrexate‐treated arthritic animals, which showed similar levels (Figure 2).

The IL‐10 immunoexpression in the synovial membrane was significantly higher among untreated arthritic animals (positive cell count mean = 63) than among either control animals (mean = 20) or methotrexate‐treated arthritic animals (mean = 23) (Figure 2), whereas the opposite was observed for the articular cartilage where control animals and methotrexate‐treated arthritic animals showed significantly higher IL‐10 expression (positive cell count mean = 211 and 172, respectively) than observed among the untreated arthritic animals (positive cell count mean = 126).

### Analysis of nociceptive behavior

**FIGURE 3:**
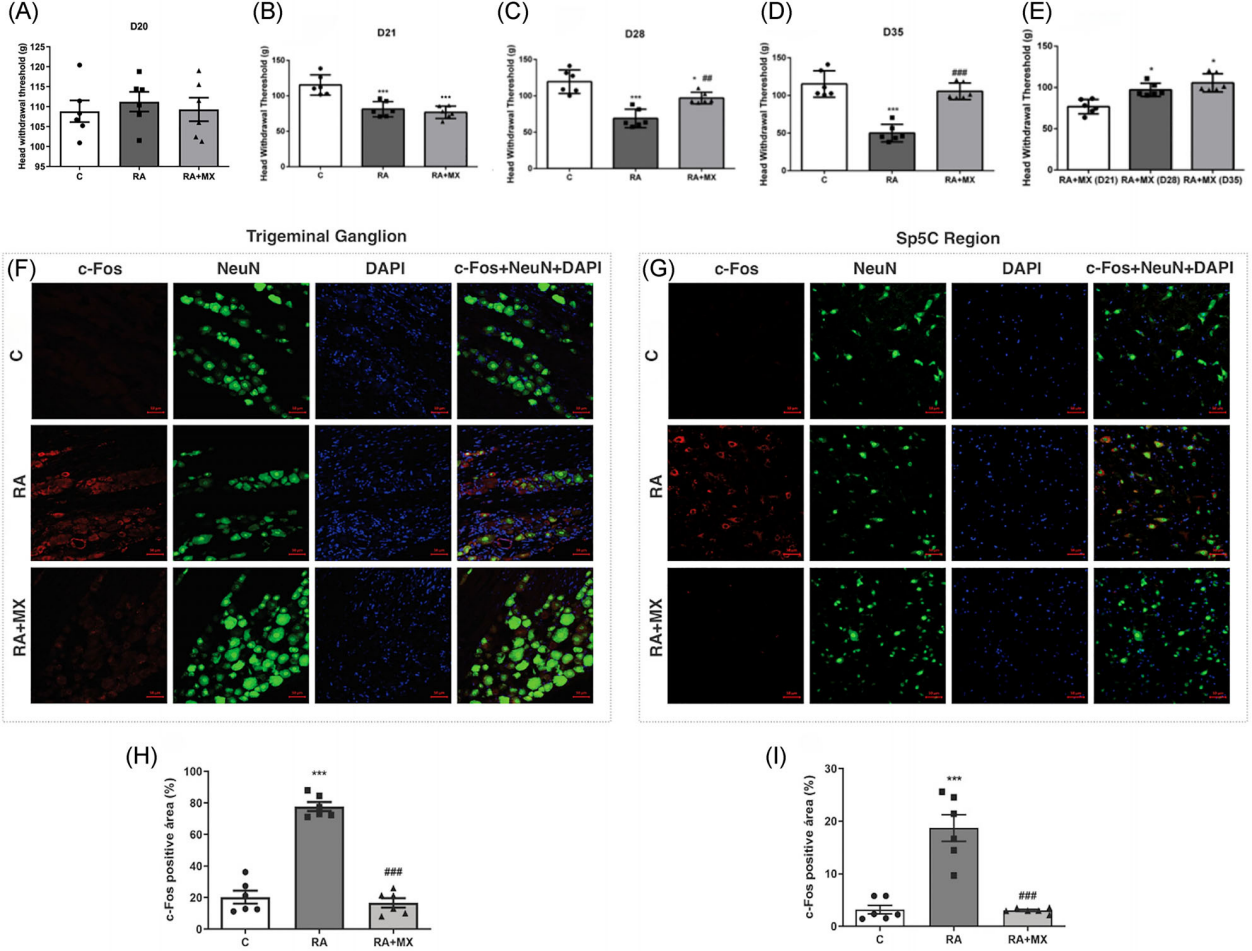
Effect of intra‐articular injection of methylated bovine serum albumin (mBSA) (10 µg) on the nociceptive response in temporomandibular joint (TMJ) arthritis model. Mechanical hyperalgesia was assessed on Days 20 (A), 21 (B), 28 (C), and 35 (D) of the experimental protocol. The nociceptive threshold in the TMJ region of arthritic animals treated with methotrexate (0.75 mg/kg; twice a week, orally) was also evaluated after the first (D21), second (D28), and third (D35) intra‐articular mBSA injections (E). c‐Fos immunoexpression (200× magnification) and quantification of positive cells (%) are shown in the trigeminal ganglion (F and H, respectively) and in the spinal trigeminal nucleus (Sp5C) region (G and I, respectively) in TMJ arthritic rats treated with methotrexate and euthanized 6 h after the third mBSA injection (*n* = 6; 200× magnification). Results are presented as mean ± standard deviation (SD) (error bars represent SD). *p < 0.05 versus control (C); **p < 0.005; ***p < 0.001 versus control (C); ##*p* < 0.01 versus RA; ###*p* < 0.001 versus RA (one‐way analysis of variance [anova], Tukey's test). Red = c‐Fos immunoexpression; green = NeuN (neuronal marker); blue 4′,6‐diamidino‐2‐phenylindole (DAPI) (nuclear marker); c‐Fos + NeuN + DAPI = merged. Sp5C region = caudal part of the spinal trigeminal nucleus. C = control group; RA = TMJ arthritic animals; RA + MX = TMJ arthritic animals treated with methotrexate.

During the development of RA, changes in mechanical hyperalgesia were observed in the left TMJ in the experimental groups. Untreated arthritic animals showed a significant decrease in the nociceptive threshold in the left TMJ over the analyzed period: compared with baseline values on D20 (before intra‐articular challenge; Figure 3A), untreated arthritic animals exhibited reduced nociceptive threshold values after the challenges on D21 (33 g reduction, *p* = 0.0003; Figure 3B), D28 (69 g reduction, *p* < 0.0001; Figure 3C), and D35 (55 g reduction, *p* < 0.0001; Figure 3D). In contrast, 6 h after the second and third intra‐articular challenges, methotrexate‐treated arthritic animals showed significantly increased nociceptive threshold values (D28: 28 g increase, *p* = 0.0045; D35: 55 g increase, *p* < 0.0001; Figure 3E). Furthermore, after the third intra‐articular challenge, there was no statistical difference between the RA + MX and C groups (Figure 3C).

Comparing the therapeutic effect of methotrexate on the days corresponding to intra‐articular administration of mBSA (D21, D28, and D35), a significantly higher nociceptive threshold was observed on D28 (21 g increase, *p* = 0.0047) and D35 (29 g increase, *p* = 0.0002) than on D21, with no statistical difference in this effect after the second and third intra‐articular injections (Figure 3D).

The evaluation of spontaneous nociceptive behavior using the Grimace scale for rats revealed that untreated arthritic animals had significantly higher scores for orbital pinching (*p* = 0.037), nose flattening (*p* = 0.015), and whisker position (*p* = 0.0014) when compared to healthy animals. Methotrexate treatment reversed these findings in arthritic animals (*p* < 0.05) (Table S2).

### Evaluation of the immunoexpression of c‐Fos, Wnt‐10b, and β‐catenin in the trigeminal nociceptive pathway

In the RA group, the induction of experimental arthritis resulted in higher immunoexpression of c‐Fos in the trigeminal ganglion (Figure 3F,H; mean positive area = 77.7%) and in the Sp5C region (Figure 3G,I; mean positive area = 18.7%) in neuronal cells when compared to the control group (mean positive area = 20.3% and 3.2%, respectively; *p* < 0.0001), suggesting peripheral and central activation of the trigeminal nociceptive pathway. Arthritic animals treated with methotrexate showed lower c‐Fos immunoexpression in this pathway when compared to untreated arthritic animals (mean in trigeminal ganglion = 18, mean in Sp5C region = 3; *p* < 0.0001) (Figure 3F–I).

**FIGURE 4:**
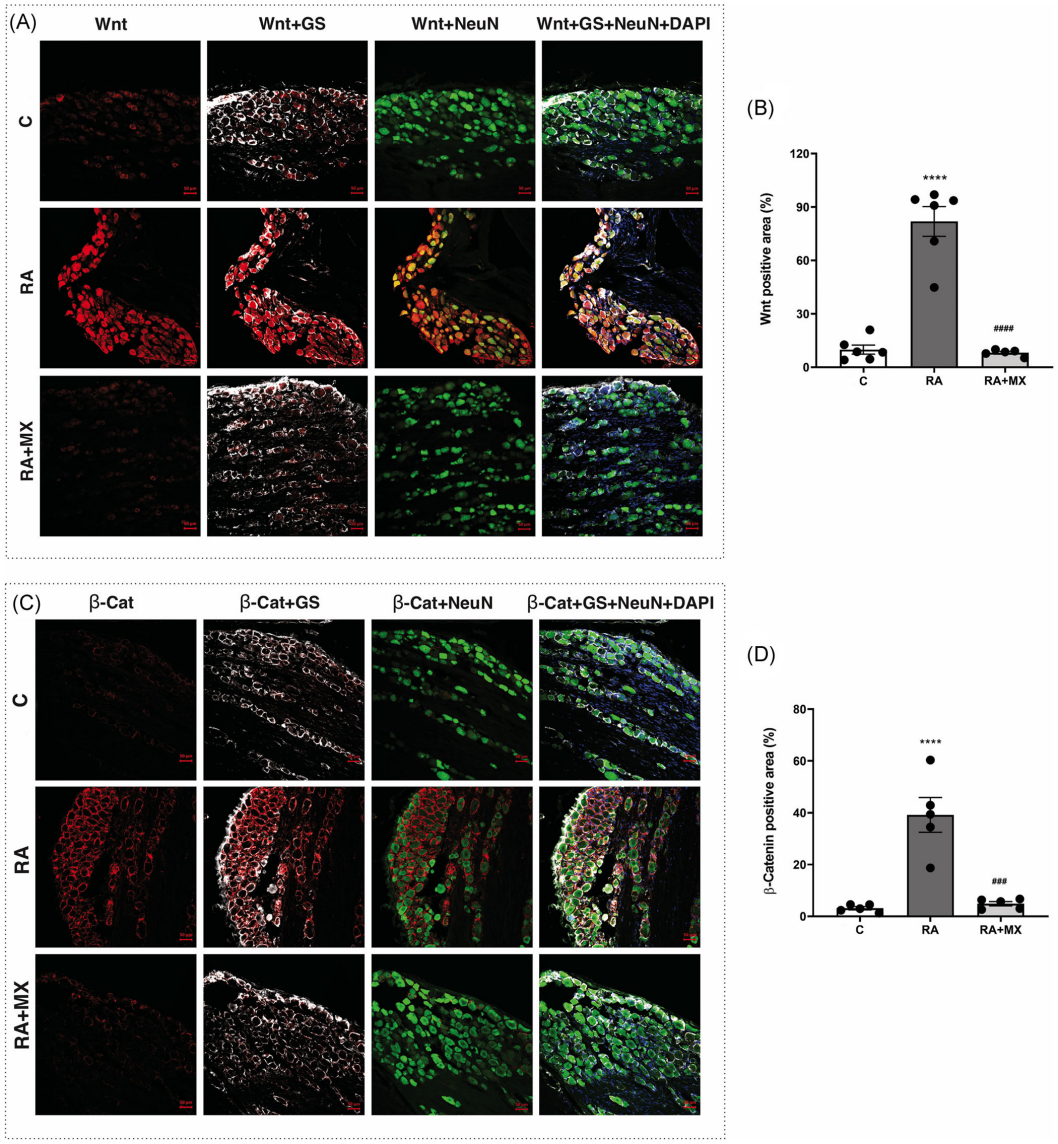
Effect of methotrexate (0.75 mg/kg; orally, twice a week) on Wnt‐10b (A) and β‐catenin (C) expression in the trigeminal ganglion of animals with temporomandibular joint (TMJ) arthritis. The immunoexpression of Wnt‐10b (B) and β‐catenin (D) in the trigeminal ganglion was evaluated by quantifying the percentage of positively labeled cells (*n* = 6; 200× magnification). Graphs are presented as mean ± standard deviation (SD) (error bars represent SD). ****p < 0.0001 versus C; ###*p *≤ 0.0001 versus RA (one‐way analysis of variance [anova]; Tukey's test). C = control group; RA = animals with TMJ arthritis; RA + MX = animals with TMJ arthritis treated with methotrexate. Wnt‐10b = Wnt; β‐cat = β‐catenin. GS = glutamine synthetase (satellite cell marker); NeuN = neuronal marker; DAPI = nuclear marker. DAPI, 4′,6‐diamidino‐2‐phenylindole.

In the RA group, higher immunoexpression of Wnt‐10b (Figure 4A,B; mean positive area = 81%, *p* < 0.0001) and β‐catenin (Figure 4C,D; mean positive area = 39.1%, *p* < 0.0001) was observed in neuronal and glial satellite cells in the trigeminal ganglion, respectively, compared to the control group (mean positive area = 9.3% and 3%, respectively) (Figure 4B,D). Arthritic animals treated with methotrexate showed lower immunoexpression of these markers (mean positive area = 7.8%, *p* < 0.0001; mean positive area = 4.8%, *p* = 0.0001, respectively) compared to untreated arthritic animals (Figure 4B,D). No immunoexpression of Wnt/β‐catenin pathway markers was observed in the Sp5C (Figure S1).

**FIGURE 5:**
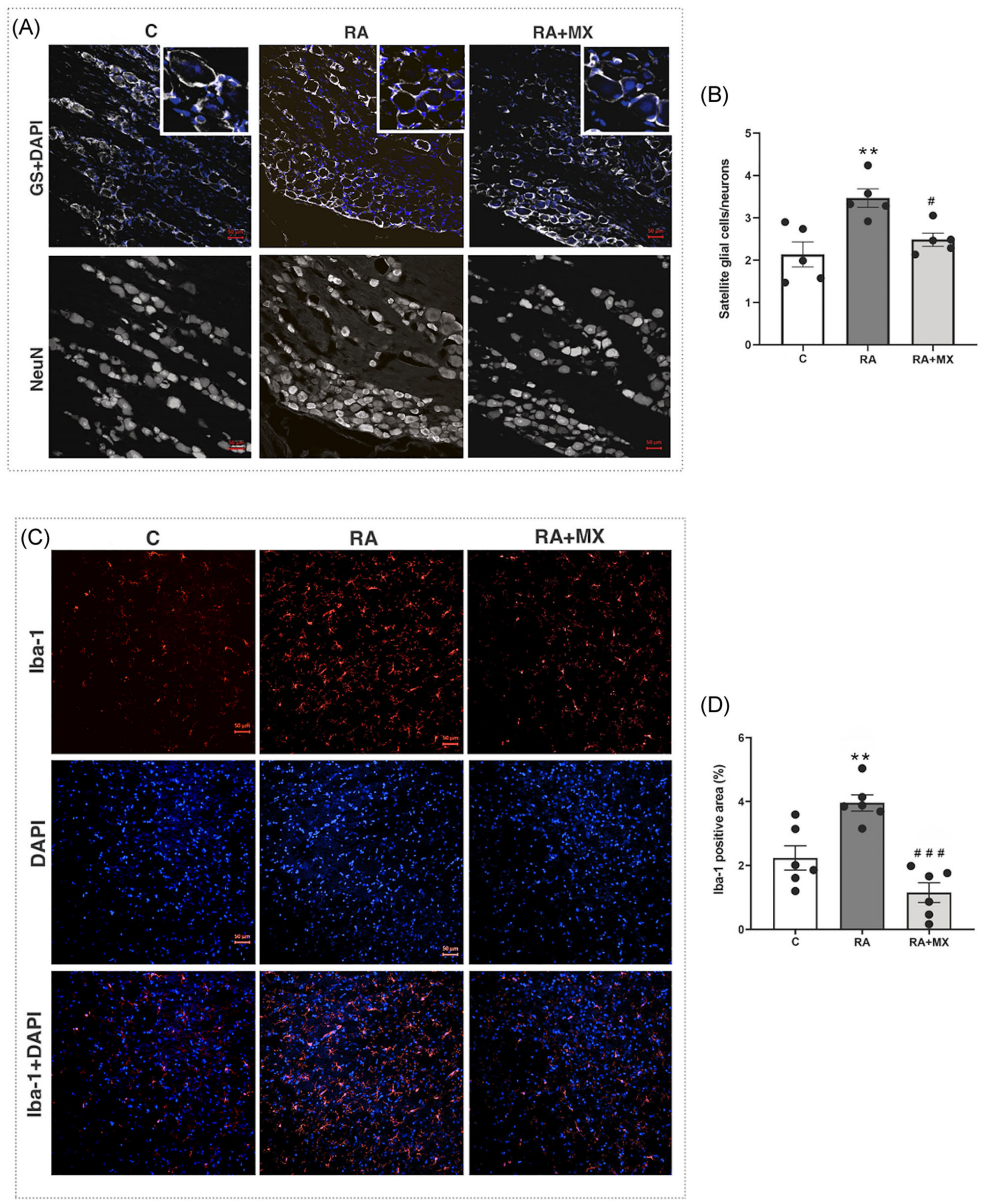
Effect of methotrexate (0.75 mg/kg; orally, twice a week) on glutamine synthetase immunoreactivity per neuron (A) and satellite cell count (B) in the trigeminal ganglion, and on Iba‐1 immunoreactive area (C) and microglia count (D) in the Sp5C region of animals with temporomandibular joint (TMJ) arthritis, 200× magnification. Results are presented as mean ± standard deviation (SD) (*n* = 6). Error bars represent SD. Satellite glial cell/neurons **p = 0.0025 versus C; #*p* = 0.011 versus RA. Iba‐1 positive area (%) ***p < 0.0001 versus C, ###*p* < 0.0001 versus RA. Sp5C region = caudal part of the spinal trigeminal nucleus. C = control group; RA = animals with TMJ arthritis; RA + MX = animals with TMJ arthritis treated with methotrexate. GS = glutamine synthetase (satellite cell marker); Iba‐1 = microglial marker; NeuN = neuronal marker; DAPI = nuclear marker. DAPI, 4′,6‐diamidino‐2‐phenylindole.

In the RA group, a higher number of satellite cells per neuron in the trigeminal ganglion (Figure 5A,B; mean positive cell count = 3.4, *p* = 0.0025) and microglia immunoexpression in the Sp5C region (Figure 5C,D; mean positive area percentage = 3.9, *p* < 0.0001) were also observed compared to the control group. Treatment with methotrexate showed a lower number of both types of glial cells than seen in the RA group (Figure 5C,D; mean positive cell count = 2.4, *p* = 0.011; mean positive area percentage = 1, *p* < 0.0001, respectively).

### Analysis of microglial/macrophage morphology

**FIGURE 6:**
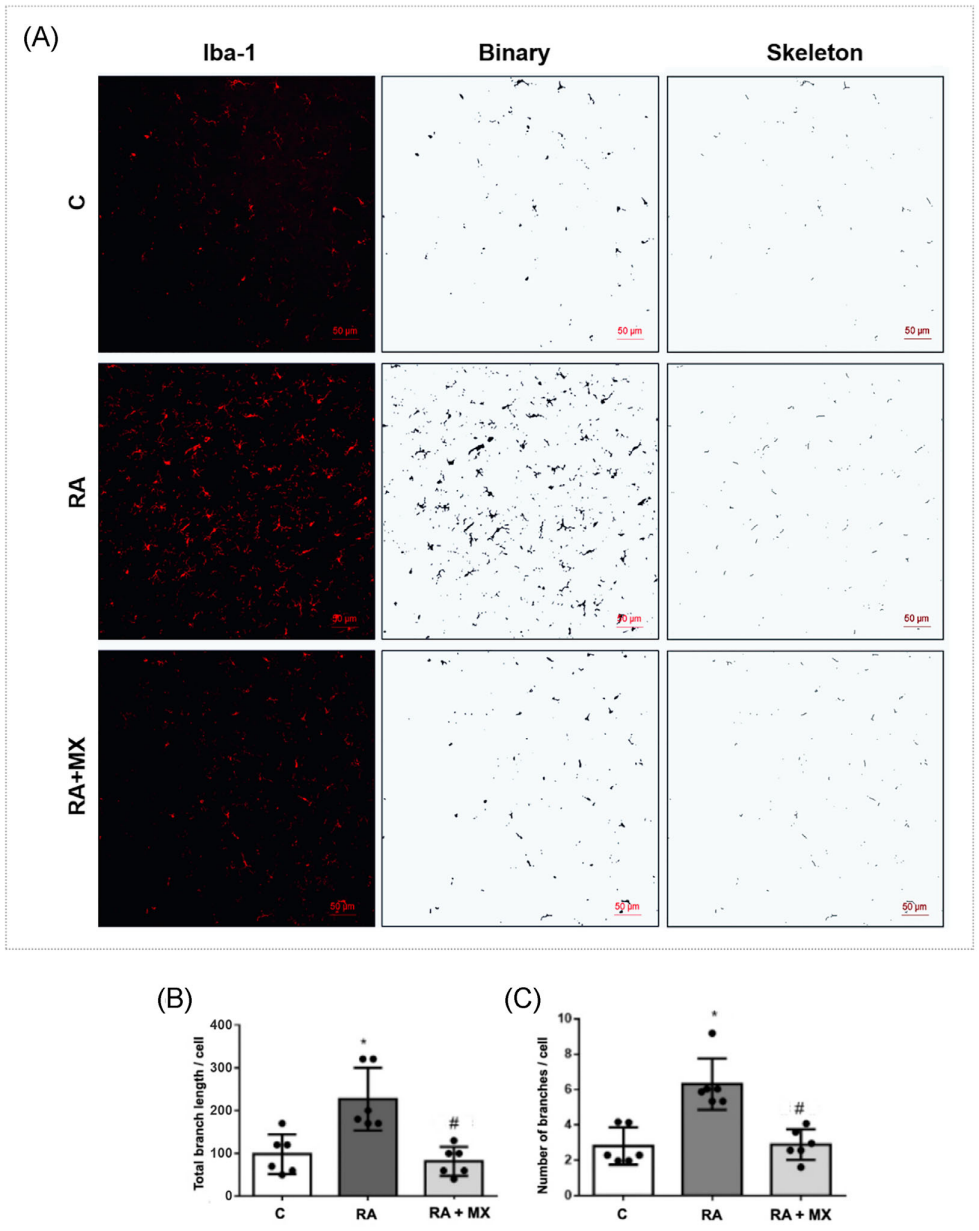
Effect of methotrexate (0.75 mg/kg; orally, twice a week) on microglial/macrophage morphology (A), total branch length (B), and number of branches per cell (C) in the Sp5C region of rats with temporomandibular joint (TMJ) arthritis. Photomicrographs acquired with maximum pinhole aperture were binarized and skeletonized; 200× magnification. Graphs are presented as mean ± standard deviation (SD) (*n* = 6). Error bars represent SD. *p < 0.05 versus C, #*p* < 0.05 versus RA. Sp5C region = caudal part of the spinal trigeminal nucleus C = control group; RA = animals with TMJ arthritis; RA + MX = animals with TMJ arthritis treated with methotrexate. Iba‐1 = microglial marker.

Methotrexate treatment caused alterations in microglial/macrophage morphology (Figure 6A). Animals with TMJ arthritis showed a higher total branch length per cell (mean = 220 µm, *p* = 0.033) and number of branches per cell (mean = 6.5, *p* = 0.01) compared with non‐arthritic animals (mean = 95 µm and 2.6 branches, respectively). Additionally, arthritic animals treated with methotrexate exhibited lower values when compared with untreated arthritic animals (mean total branch length = 70 µm, *p* = 0.032; mean number of branches = 3.4, *p* = 0.029, respectively). No statistical differences were found between the control and RA + MX groups (Figure 6B,C).

## DISCUSSION

Our study demonstrated that methotrexate treatment in animals with TMJ arthritis effectively reduced mechanical hyperalgesia, inflammation, and joint damage. It also decreased the immunoexpression of c‐Fos, Wnt‐10b, and β‐catenin in the trigeminal nociceptive pathway, as well as glutamine synthetase and Iba‐1 in glial cells. Furthermore, a significant reduction was observed in the total length and branching of microglial/macrophage cells.

Thus, when comparing untreated arthritic animals with those treated with methotrexate, clear differences were observed. In treated animals, methotrexate, beyond its classical anti‐inflammatory and immunosuppressive actions, appeared to modulate Wnt/β‐catenin signaling. These findings suggest that the therapeutic effects of methotrexate may include not only the suppression of inflammation but also a regulatory impact on Wnt/β‐catenin‐mediated pathways, thereby contributing to the attenuation of both joint damage and nociceptive sensitization.

Methotrexate, a disease‐modifying antirheumatic drug commonly prescribed for managing various forms of arthritis, including rheumatoid arthritis, exerts its anti‐inflammatory effects by reducing neutrophil recruitment and the production of pro‐inflammatory mediators [9]. Low doses of methotrexate are typically indicated for treating joint pain and inflammation. However, it is important to note that higher doses have been associated with degenerative changes in the TMJ of rats [14]. In an experimental model of TMJ arthritis in rabbits, induced by intra‐articular administration of ovalbumin, methotrexate reduced inflammatory parameters but failed to completely eliminate the signs of arthritis [15]. In our study, methotrexate treatment alone not only alleviated joint damage but also reduced inflammatory parameters.

Although the anatomical and histological organization of the TMJ offers a greater capacity for adaptation and repair, it remains susceptible to the molecular mechanisms driving the pathogenesis of rheumatoid arthritis [5]. In addition to these histopathological changes, rheumatoid arthritis is characterized by dysregulation of the host's innate and adaptive immune systems, leading to altered responses of B cells, Th1, Th17, and T regulatory cells, and consequently affecting the synthesis of pro‐inflammatory and anti‐inflammatory mediators [16]. Following arthritis induction, an increased immunoexpression of TNF‐α and IL‐17 was observed in the synovial membrane and articular cartilage. This response involves both immune and non‐immune cells in the joint environment. Synovial fibroblasts and chondrocytes can become immunoreactive when exposed to inflammatory diseases [6, 9]. Notably, methotrexate treatment reduced their immunoexpression in our study. TNF‐α is associated with the hyperexcitability of sensory neurons and can directly stimulate nociceptors, playing a significant role in enhancing pain sensitivity [17]. IL‐17 plays a key role in the progression of rheumatoid arthritis in the TMJ, amplifying inflammation by stimulating the release of other pro‐inflammatory cytokines (such as TNF‐α, IL‐6, and IL‐1β), chemokines, and matrix metalloproteinases [18].

IL‐10 plays a controversial role in inflammatory and autoimmune disorders. IL‐10 deficiency is associated with increased tissue damage [19], and in diseases like rheumatoid arthritis, an endogenous immunological control mechanism enhances IL‐10 production, promoting repair‐related events and reducing the sensitization of sensory nerve endings [20]. IL‐10 is abundantly expressed in the synovial fluid of patients with rheumatoid arthritis. A previous study demonstrated that IL‐10 produced by non‐T cells in RA patients contributes to the suppression of T cell function and the increased production of autoantibodies and rheumatoid factor [21]. Greenhill *et al.* [22] showed that IL‐10 knockout mice exhibit enhanced bone damage and focal bone erosions. In our study, methotrexate treatment increased IL‐10 immunostaining in the articular cartilage, which may be linked to an attempted repair process in the joint.

In the absence of treatment, the ongoing peripheral inflammatory process in the joint leads to chronic pain. This condition results from neuroimmune interactions involving neurons, glial cells, and inflammatory cells, ultimately leading to central sensitization. Peripheral sensitization plays a critical role in the transition from acute to chronic pain, while central sensitization contributes to the persistence of pain, affecting emotional and affective aspects as well. A previous study found that a reduction in the nociceptive threshold is associated with inflammation and joint damage in the TMJ, as well as increased expression of c‐Fos [6]. Notably, treatment with methotrexate reduced the immunoexpression of c‐Fos, suggesting that methotrexate decreases neuronal activity throughout the trigeminal nociceptive pathway.

Our findings demonstrate a significant increase in the number and complexity of microglial/macrophage processes in the Sp5C region of rats with TMJ arthritis, along with an increased number of satellite glial cells surrounding neurons in the trigeminal ganglion. These morphological alterations are indicative of glial activation and are consistent with previous reports linking glial responses to central sensitization and the maintenance of chronic pain states [23, 24]. Notably, methotrexate treatment reversed these changes, suggesting that it exerts a modulatory effect on glial reactivity in this model.

Although microglial/macrophage morphology has traditionally been used to infer functional states, particularly the transition from a ramified to an amoeboid form as a hallmark of activation, we recognize that morphology alone may not fully capture the functional diversity of microglial/macrophage responses [25]. In fact, hyper‐ramification may itself represent a reactive state, particularly in the context of chronic or low‐grade inflammation. In our study, the observed increase in microglial branching and total process length in the Sp5C may reflect such a primed or sensitized state in response to sustained inflammatory input [26]. The reversal of these changes following methotrexate treatment supports the interpretation that this morphological profile is dynamically regulated and potentially reversible.

By integrating morphometric assessment with regional glial and infiltrating macrophage quantification, our findings contribute to the growing understanding of glial plasticity in TMJ inflammation. Nevertheless, future studies could help more comprehensive, context‐specific strategies to assess microglial function, such as functional marker expression, live imaging, or transcriptomic profiling—to more precisely define their roles in orofacial inflammatory pain.

Several pharmacological agents have been shown to suppress the development of neuropathic pain by inhibiting glial activation and, consequently, reducing the release of pro‐inflammatory cytokines involved in pain chronification [27, 28]. For example, diclofenac, a non‐steroidal anti‐inflammatory drug, and gabapentin, a widely used treatment for neuropathic pain, were both shown to reduce pain‐like behavior during the inflammatory phase; however, only gabapentin maintained this effect during the late phase [29]. Interestingly, although high systemic doses of methotrexate have been associated with microgliosis, microglial activation, and cognitive impairment in rats [30], low‐dose intrathecal administration was found to attenuate microglial activation, decrease p38 phosphorylation, and improve pain‐like behavior in a model of neuropathic pain induced by chronic constriction injury [28].

Previous studies have demonstrated that abnormal expression of Wnt and β‐catenin ligands in the joints or nociceptive pathways may be associated to neuroinflammation and pain perception in experimental models of persistent arthritis [5, 6, 31]. Thus, studying neuron‐glia interaction regulated by the canonical Wnt pathway may help elucidate the neuroinflammatory mechanisms in RA and offer new perspectives for pain management strategies. Research has shown that positive regulation of this signaling pathway contributes to the pathogenesis of various chronic pain and neuropathic conditions by modulating nervous system activity [32, 33]. Furthermore, females exhibit a higher inflammatory response when compared to males, which may influence in initiation and maintenance of a painful state [34, 35].

In a rat model of TMJ osteoarthritis, this signaling pathway was associated with the progression of mandibular condylar cartilage degeneration and subchondral bone loss [36]. In a previous study from our group, TMJ arthritis was shown to increase the immunoexpression of Wnt‐10b and β‐catenin in the cartilage, synovial membrane, and TG. In addition, downstream mediators such as cyclin D1 and c‐Myc were also upregulated in the arthritic TMJ [6]. These molecular changes were accompanied by elevated levels of proinflammatory cytokines (IL‐1β, TNF‐α, IL‐6) and reduced nociceptive thresholds, indicative of peripheral sensitization. The increased immunoexpression of Wnt‐10b in sensory neurons and β‐catenin in glial satellite cells in the TG of rats with TMJ arthritis further reinforces the involvement of the Wnt/β‐catenin pathway in the nociceptive mechanisms of rheumatic diseases [6].

Arthritic animals treated with methotrexate exhibited a reduction in the immunoexpression of Wnt‐10b in peripheral sensory neurons and β‐catenin in glial satellite cells. It is important to note that no immunoexpression of these components of the canonical Wnt pathway was observed in the Sp5C region. However, to confirm that this signaling pathway does not participate in central sensitization, further studies are needed to evaluate the chronic phase of the disease.

In conclusion, the treatment with methotrexate in rats with TMJ arthritis reduced pain, inflammation, and joint damage, as well as immunoexpression of the canonical Wnt pathway and neuronal and glial cell activation in the trigeminal nociceptive pathway.

## AUTHOR CONTRIBUTIONS

**Conceptualization**: Ana Carolina de Figueiredo Costa, Luane Macedo de Sousa, Delane Viana Gondim. **Methodology**: Ana Carolina de Figueiredo Costa, Luane Macedo de Sousa, Paula Goes, Mariana Lima Vale, Delane Viana Gondim. **Investigation**: Ana Carolina de Figueiredo Costa, Luane Macedo de Sousa, Sofia Tavares Bessa, Anamaria Falcão Pereira, Paula Goes, Delane Viana Gondim. **Formal analysis**: Ana Carolina de Figueiredo Costa, Sofia Tavares Bessa, Anamaria Falcão Pereira. **Data Curation**: Ana Carolina de Figueiredo Costa, Sofia Tavares Bessa, Anamaria Falcão Pereira. **Resources**: Delane Viana Gondim, Mariana Lima Vale. **Supervision**: Delane Viana Gondim, Paula Goes. All authors read and approved the manuscript.

## CONFLICT OF INTEREST STATEMENT

The authors declare no conflicts of interest.

## Supporting information



Supporting Information
