# Supplementary material for: Effects of methotrexate on Wnt/β‐catenin signaling and glial activation in temporomandibular joint arthritis in rats
Source: Eur J Oral Sci. 2025 Sep 26;133(6):e70041. doi: 10.1111/eos.70041 (PMC12628117; doi:10.1111/eos.70041)

SUPPORTING INFORMATION

Effects of methotrexate on Wnt/β-catenin signaling and glial activation in temporomandibular joint arthritis in rats

COSTA ACF, DE SOUSA LM, BESSA ST, PEREIRA AF, GOES P, VALE ML, GONDIM DV.

Faculty of Pharmacy, Dentistry and Nursing; and

Faculty of Medicine, Federal University of Ceará, Fortaleza, CE, Brazil.

Table S1 –Analysis of inflammatory infiltration in synovial membrane and joint damage in the temporomandibular joint (TMJ) of arthritic rats treated with methotrexate (0.75 mg/kg; twice weekly, orally). Both parameters were scored on a scale from 0-3, and the data presented are the median and the minimum and maximum scores.

**TMJ Arthritis Parameters (n=6)**	**Control rats**	**Rats with TMJ arthritis**	**Rats with TMJ arthritis treated with methotrexate**
Synovial membrane	0 (0-0)	3 (2-3)*	1 (1-2)
Articular cartilage	0 (0-1)	2 (1-2)*	0 (0-2)[#]

* p <0.05 *versus* Controls, # p= 0.02 *versus* rats with TMJ arthritis; Kruskal-Wallis; Dunn.

Table S2. Analysis of facial expressions in rats with temporomandibular joint (TMJ) arthritis treated with methotrexate. Values presented are the median (min-max) scores according to the Grimace Rat Scale.

Parameters (n=6)	Control rats	Rats with TMJ arthritis	Rats with TMJ arthritis treated with methotrexate
**Orbital pinching**	0 (0-1)	1 (1-2)*	0 (0-1)#
**Nose flattening**	0 (0-1)	2 (1-2)*	0 (0-1)#
**Ear changes**	0 (0-0)	1 (0-2)	0 (0-1)
**Whisker change**	0 (0-0)	1.5 (1-2)**	0 (0-0)##

* p <0.05 *versus* Controls, ** p= 0.001 *versus* Controls, # p<0.05 *versus* TMJ arthritis, ## p=0.001 versus TMJ arthritis (Kruskal-Wallis; Dunn).

Figure S1. Effect of methotrexate (0.75 mg; orally, twice a week) on Wnt10b and β-catenin expression in the Sp5C region of animals with TMJ arthritis. No immunoexpression of Wnt10b or β-catenin was observed in the Sp5C region of animals with TMJ arthritis, regardless of methotrexate treatment.

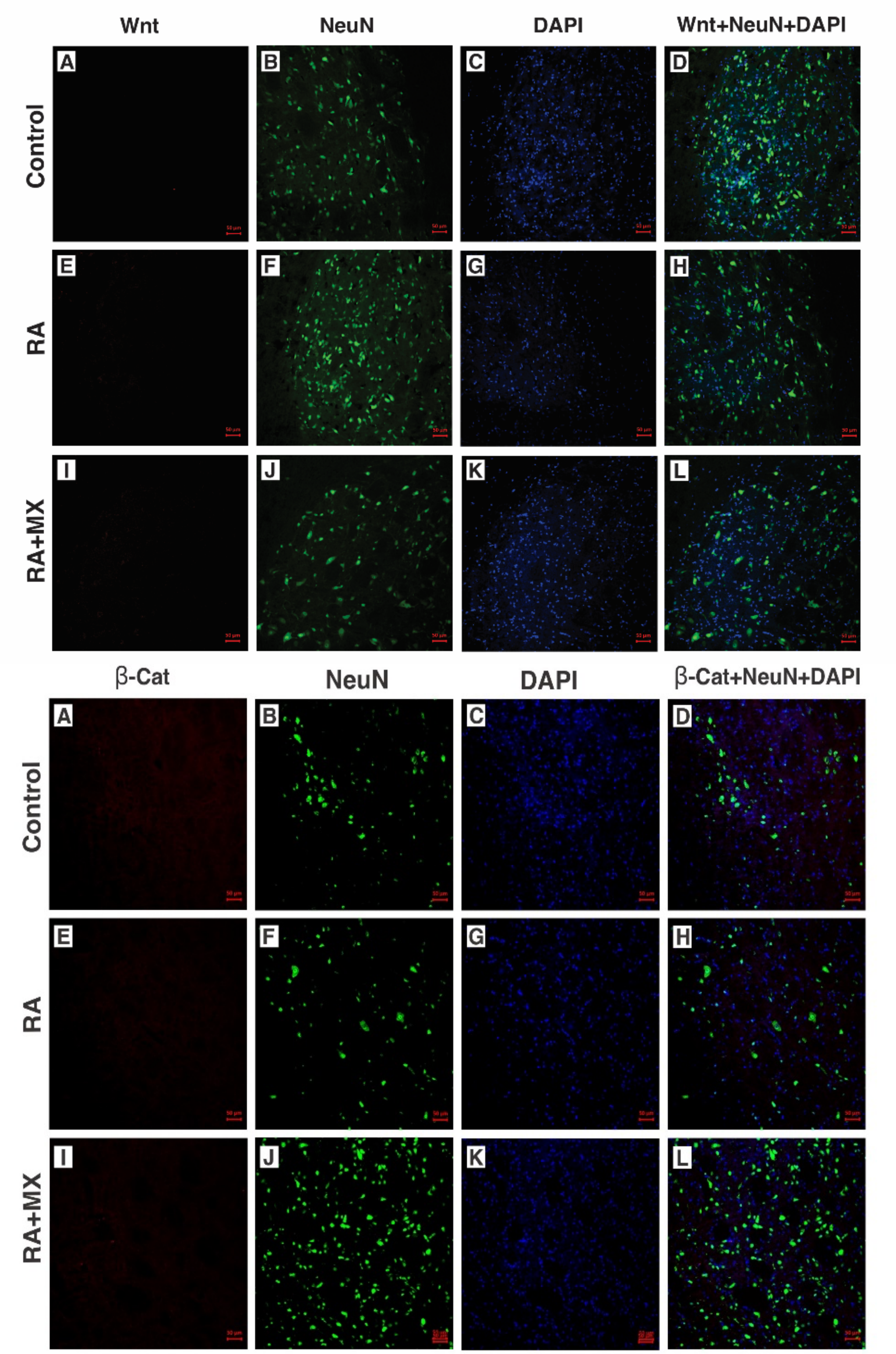

Supplement: Supplementary file 1 — Supporting Information [file EOS-133-e70041-s001.pdf]
